# Genetic polymorphism of *scrA* gene of *Streptococcus mutans* isolates is not associated with biofilm formation in severe early childhood caries

**DOI:** 10.1186/s12903-017-0407-0

**Published:** 2017-07-14

**Authors:** Yan Zhou, Lixia Yu, Ye Tao, Qinghui Zhi, Huancai Lin

**Affiliations:** 1 Department of Preventive Dentistry, Guanghua School of Stomatology, Sun Yat-Sen University, 56 Ling Yuan Road West, Guangzhou, 510055 China; 2 Guangdong Provincial Key Laboratory of Stomatology, Sun Yat-Sen University, Guangzhou, 510055 China

**Keywords:** *Streptococcus mutans*, Biofilm, *scrA* gene, Caries

## Abstract

**Background:**

To explore and analyse the association between biofilm and the genetic polymorphisms of *scrA* gene of EnzymeIIscr found in clinical isolates of *Streptococcus mutans* (*S. mutans*) from severe early childhood caries (S-ECC) in 3 years old children.

**Methods:**

Clinical strains of *S. mutans* were conserved from a previous study. Thirty strains of *S. mutans* from the S-ECC group and 30 strains of *S. mutans* from the caries free (CF) group were selected. Biomass and viability of biofilm formed by the strains were evaluated by crystal violet and alamar blue assay. Genomic DNA was extracted from the *S. mutans* isolates. PCR was conducted to amplify *scrA* gene. After purified and sequenced the PCR products, BioEdit sofeware was used to analyse the sequence results. A chi-square test was used to compare the results.

**Results:**

Compared to the CF group, the biomass of S-ECC group was higher (*P* = 0.0424). However, the viability of the two groups showed no significant difference. All 60 clinically isolated *S. mutans* strains had a 1995 base pair (bp) *scrA* gene. Forty-nine point mutations were identified in *scrA* from the 60 clinical isolates. There were 17 missense point mutations at the 10, 65, 103, 284, 289, 925, 1444, 1487, 1494, 1508, 1553, 1576, 1786, 1822, 1863, 1886, and 1925 bp positions. The other 32 mutations were silent point mutations. No positions were found at active sites of ScrA. The statistic analyse showed no significant missense mutation rates between the two groups.

**Conclusions:**

There was no association between biofilm and genetic polymorphisms of *scrA* from *S. mutans* with S-ECC in 3 years old children.

**Electronic supplementary material:**

The online version of this article (doi:10.1186/s12903-017-0407-0) contains supplementary material, which is available to authorized users.

## Background

Severe early childhood caries (S-ECC) is a serious oral public health problem in the world. Drury et al gave a brief definition of S-ECC [1]. They regarded any sign of smooth-surface caries in children younger than 3 years old as S-ECC. From ages three through five, one or more cavitated, missing (due to caries), or filled smooth surfaces in primary maxillary anterior teeth or a decayed, missing, or filled score of greater than or equal to four (age 3), greater than or equal to five (age 4), or greater than or equal to six (age 5) surfaces constitutes S-ECC [1].

S-ECC is an infectious disease, with bacteria as the important causative agent. Sucrose plays a key role in the development of this infection. *Streptococcus mutans* (*S. mutans*) bacteria have been identified as the primary agent in the pathogenic mechanism of dental caries [2]. Sugar metabolism by the acid-forming *S. mutans* is directly related to the development of dental caries. In the process of metabolism of sucrose, sucrose is transported by the phosphoenolpyruvate:sugar phosphotransferase (PTS) system [3]. Each PTS consists of three main constituents: enzyme I (EI), a heat-stable protein (HPr), and enzyme II (EII). The *scrA* gene encodes Enzyme II of *S. mutans* [4]. The regulation of EII^scr^ expression and activity should play an important role in the ability of *S.mutans* to demineralize human teeth [4]. A novel regulatory circuit has been reported that *scrA* served as a central role for the control of sucrose catabolism [5]. These indicate that *scrA* gene is very important in the cariogenicity of *S. mutans*, thus having an effect on the susceptibility of dental caries.

Though there is a strong relationship between *S. mutans* and S-ECC, children colonized by *S. mutans* do not all apparent S-ECC [6]. It has been proposed that *S. mutans* isolates from S-ECC are genetically distinct from caries free (CF) children [7]. The ability to form a biofilm probably differs among clinical strains.

As *scrA* plays a central role in sucrose catabolism, we hypothesized that it have probably been genetic polymorphisms in clinical strains of *S. mutans* that impact the ability to form biofilms. The purpose of this communication is to describe the association between biofilm and the genetic polymorphisms of the *scrA* gene of Enzyme II found in clinical strains of *S. mutans* from S-ECC in 3 years old children.

## Methods

### Sample collection

Subjects were participants in a previous study. The study conducted in Guangzhou, southern China. It was a case-control study, which has been previously described in detail [8]. Briefly, dental plaque samples were collected from 3-year-old children. These children were recruited from nursery schools in a suburb of Guangzhou. Mixtures of dental plaque were taken from the labial/buccal surfaces of maxillary teeth by using sterile cotton swabs. The cut cotton swabs were put in a sterile fluid thioglycolate medium immediately and transferred to the laboratory on ice within 4 h. This stuy obtained ethical approval from an ethics committee of Sun Yat-sen University (Number is ERC-[2012]-13).

### Bacterial strains

Plaque samples were mixed and dispersed to obtain a dilution series to 10^−3^ dilutions., Brifely, we prepared Mitis-Salivarius-Bacitracin (MSB) agar with 20% sucrose and 0.2 units/ml bacitracin. 50 μl of the diluent was plated onto Mitis-Salivarius-Bacitracin (MSB) agar, and incubated anaerobically (85% N_2_, 5% CO_2_, and 10% H_2_) at 37 °C for 72 h [9]. According to the colony morphology, we randomly selected two colonies from each child The *S. mutans* strains(ATCC700610/UA159, Guangdong culture collection center,China) were grown in brain heart infusion broth (BHI, Huankai microbial, China) anaerobically. (10%H_2_, 10%CO_2_, 80%N_2_). The reference strain was *S. mutans* UA159. Next, the ability to ferment mannitol, sorbitol, raffinose, melibiose, and aesculin and to hydrolyse arginine of colonies were tested [10]. The identified strains were streaked onto MSB agar. Pure strains were preserved in 50% glycerol at -80 °C. From the *S. mutans* positive children, we picked 30 children from the S-ECC group and 30 children from the CF group. In total, sixty isolates of *S. mutans* from S-ECC children and CF children were used in the next step.

### Biomass and viability of strains

*S. mutans* was incubated in BHI with 1% sucrose in 96 well flat-bottom microtitre plate (Corning Incorporated, NY, USA) for 24 at 37 °C in a 5% CO_2_ incubator. After that, biofilms of strains were carefully washed twice with PBS, and biofilm biomass was determined using the crystal violet (CV) assay described by Sabaeifard [11]. Viability of biofilms formed by strains was evaluated by the Alamar Blue® assay [12]. The method is based on the dye resazurin. Reducing molecules derived from bacterial metabolism converted resazurin to the fluorescent molecule resorufin. which is converted to the fluorescent molecule resorufin by. The percentage reduction in biofilm viability was calculated according to the manufacturer’s instructions.

### DNA extraction

The clinical isolates were incubated in 2 ml of BHI broth overnight at 37 °C. When the isolates reached stationary phase, the liquid was centrifuged at 12 000 rpm for 5 min. The supernatant was discarded and the remaining cells were washed with phosphate buffered saline(PBS) for twice.. We used a Qiagen DNA mini kit to extract DNA from samples (Qiagen, Germany). The DNA concentration and purity were determined spectrophotometrically by measuring the A260 and A280 (Varian, USA). DNA samples were stored at –80 °C until required. Genomic DNA from *S. mutans* UA159 was used as the reference.

### Amplification of *scrA* gene

The total length of *scrA* gene was 1995 bp. It was localized in the 1739208-1741202 bp position of UA159. All primers used for PCR were designed by Primer Express 2.0 software according to the UA159 *scrA* gene. As *scrA* is too long to amplify in one reaction, we designed five primers to amplify the whole *scrA* fragment [Table 1].

**Table 1:** Primer sequence used for detection of of *scrA* gene in *S. mutans*

Name	Sequence	Product length
1F	5′-CTTGATAGCGGCGATATCTG-3′	
1R	5′-TTAAGAGACCGCCTGCTACC-3′	616
2F	5′-ATTGCTGCCAGTGGTAAAAAG-3′	
2R	5′-CTGCTGAGGGCAATCTCTTATG-3′	522
3F	5′-AATATTTTTGGGTTGCATGTTAC-3′	
3R	5′-GCACTAGCTGAGCCAATCAGA-3′	589
4F	5′-GCAGCAACCTTTGCAATTTAC-3′	
4R	5′-TCAACCGGTGCATAAACTGT-3′	574
5F	5′-ATGAAGTTCTTGCGGCTCCT-3′	
5R	5′-GCCAAAAGGCTTTAATACTATTGT-3′	532

The total reaction volume of PCR amplification was 25 μl. The samples were preheated of 5 min at 95 °C. followed by 35 cycles of 30s at 95 °C, 30 s at 60 °C and 45 s at 72 °C. A final elongation step of 5 min.at 72 °C Amplified product was electrophoresed in a 1.5% (wt/vol) agarose gel. A molecular size marker (Takara, Japan) was electrophoresed in parallel.

### Sequencing of *scrA* gene

A Qiaquick Gel Extraction Kit (QIAgen, Hilden, Germany) was used to purify the PCR products. Bidirectional sequencing of all amplified product were carried by Life Technologies Company (Shanghai, China). The results of sequence were aligned with known *scrA* sequences of UA159(GenBank accession number NC_004350.2). BioEdit sofeware was used to analyse the sequence results.

### Statistical analysis

Student *t*-test was used to determine statistically significant difference of biomass and viablilty between the S-ECC and the CF group. A chi-square test was used to measure different *scrA* sequences between the groups. Statistical significance was achieved if a *P*-value below 0.05.

## Results

### Biomass and viability of strains

**Fig. 1:**
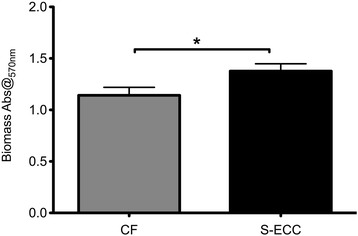
Biofilm formation of *Streptococcus mutans* isolates from caries free group and severe early childhood caries group. *Streptococcus mutans* isolates were incubated on 96-well flat-bottom plates in 1% sucrose BHI media for 24 h. The biomass of *Streptococcus mutans* isolates from caries free (CF) group and severe early childhood caries (S-ECC) group were evaluated by crystal violet assay. The results showed that isolates from S-ECC group have a higher biomass than CF group

All the isolates formed biofilm on 96 flat plates. The mean values of biomass of S-ECC group was higher than CF group (1.38 *v.s.* 1.14). The *p* values was 0.0424 (Fig. 1). The percentage reduction in biofilm viability was calculated. The values of S-ECC group *v.s.* CF group was 40.24% *v.s*. 39.19%. The percentage of two groups showed no statisticlly significant difference (*p* = 0.8156).

### PCR products

The five DNA fragment carrying the partial *scrA* gene was amplified from the sixty *S. mutans* isolates by PCR (Additional file 1). Gel electrophoresis showed a single positive band for those PCR reactions (Additional file 2).

### Sequencing results

The PCR fragments of *scrA* genes from the 60 *S. mutans* isolates were sequenced. Sequence results showed that all the clinical strains can amplify the *scrA* gene. Seven clinical strains of them had base deletion located in 1476-1487. Two strains had deletion in S-ECC group while 5 strains had deletion in CF group. No statistically significant difference were found between the two groups by using chi-square test (*P* = 0.228).

**Fig. 2:**
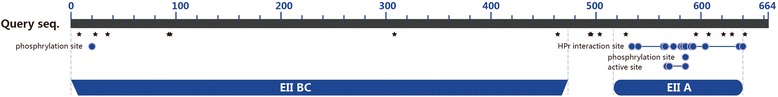
A sketch map to illustrate i) active site; ii) phosphorylation site; iii) Hpr interaction site; iv) different domains (EIIA, EIIB, EIIC) of UA159 (see *blue dot*). The missense mutations (see *black asterisk*) we found are not located in any above specific domains

The results of sequencing showed 49 mutation loci among the 60 clinical strains. The number of silent mutations was thirty-two while the number of missense mutations was seventeen. There were missense point mutations at the 10, 65, 103, 284, 289, 925, 1444, 1487, 1494, 1508, 1553, 1576, 1786, 1822, 1863, 1886, and 1925 bp positions (Fig. 2 & Additional file 3). Table 2 showed the transition of amino acids according to codon.

**Table 2:** Transversion of amino acid due to missense mutations according to codons

Base site	UA159		Clinical isolates	
	Codon	Amino acid	Codon	Amino acid
10	AGC	serine	GGC	glycin
65	GCC	alanine	GTC	vlaine
103	GAT	asparagine	AAT	asparagine
284	GCC	alanine	GTC	vlaine
289	GGT	glycin	AGT	serine
925	ACA	threonine	GCA	alanine
1444	GTC	vlaine	ATC	isoleucine
1487	GTG	vlaine	GCG	alanine
1494	GAA	glutamic acid	GAT	asparagine
1508	GCT	alanine	GTT	vlaine
1553	GCG	alanine	GTG	vlaine
1576	GTT	vlaine	ATT	isoleucine
1786	AAA	lysine	GAA	glutamic acid
1822	ATT	isoleucine	GTT	vlaine
1863	AAA	lysine	AAG	lysine
1886	AAT	asparagine	AGT	serine
1925	GCG	alanine	GTG	vlaine

The frequency of missense mutation loci of the *S. mutans* isolates was listed in Table 3. The distribution of the missense showed no statistical difference between the two groups.

**Table 3:** Analysis of the missense mutation rates in relation to caries status

Codon	S-ECC (n)	CF (n)	*x* ^2^	*P-* values
10 A → G^a^	28	28	—	1.000*
65 C → T	2	0	—	0.492*
103 G → A	1	0	—	1.000*
284 C → T	1	1	—	1.000*
289 G → A	1	0	—	1.000*
925 A → G	5	7	0.417	0.519
1444 G → A	2	0	—	0.492*
1487 T → C	20	21	0.077	0.781
1494 A → T	1	1	—	1.000*
1508 C → T	3	1	—	0.612*
1553 C → T	1	1	—	1.000*
1576 G → A	1	1	—	1.000*
1786 A → G	1	1	—	1.000*
1822 A → G	28	24	—	0.254*
1863 A → G	28	24	—	0.254*
1886 A → G	1	1	—	1.000*
1925 C → T	1	0	—	1.000*

* A chi-square test

^a^A → G, A represents the 10 locus base in UA159, G represents the 10 locus base in the clinical strains

## Discussion

In *S. mutans*, sucrose can be internalized by multiple enzymes. Enzymes include PTS, the multiple-sugar metabolism (Msm) system [13] and the maltose/maltodextrin ATP-binding cassette transporter [14]. Zeng et al. manipulated several mutans lacking one or two sucrolytic pathways to explpre the mechansism of sucrose catabolism. The results showed *scrA* gene of sucrose-PTS played a central role in regulation of exopolysaccharide metabolism [5].

The present study showed that all the isolates could form biofilms in 1% sucrose, which confirmed the important role of sucrose in the formation of biofilm in clinical isolates. The results showed that the S-ECC group had a greater ability to form biofilms. Mature biofilm is composed of bacteria and extracellular matrix. Bacteria-derived extracellular matrix is a critical virulence determinant in *S. mutans* biofilms [15]. The results agree with previous research, supporting the notion that the diversity of biofilm formation of *S. mutans* isolates may have important implications for understanding the different cariogenic ability of isolates from children.

Next, the mechanism of the diversity of biofilm formation between isolates was studied by sequencing the *scrA* gene. *ScrA* plays a very crucial role in the metabolism of sucrose [5]. Sequencing results showed that all clinical strains of *S. mutans* do have *scrA,* and that they have point mutations. However, the rates of missense mutation between two groups revealed no significant difference.. Among the 17 missense point mutation positions, there was no positions located in the enzyme –activity sites of *scrA*.

The genomes of *S. mutans* encode as many as 15 EII permeases in few strains. These EII permeases consist of different domains, including A, B, C, and D domains. Most strains of *S. mutans* possessed these permeases, but some strains harboured a few permeases [16]. It was reported that a new sucrose utilization related PTS^Bio^ transport system was identified [17]. Though there is evidence that the carbohydrates are transported via PTS activity, many other enzymes have been involved in the formation of biofilm by *S. mutans..* Successful of biofilm formation by *S. mutans* on the surface of the teeth is closely related to the activity of glucosyltransferases (GTFs) [18]. In addition, a fructosyltransferase (FTF) enzyme produced fructans from sucrose, which serve mainly as an extracellular storage polymer [19].

Clearly, great progress has been made on understanding the complexities of biofilm formation in clinical isolates. Further work will be undertaken to fully understand the mechanism of heterogeneity of *S. mutans* isolates.

## Conclusions

The present study suggest that the heterogeneity of biofilm in *S. mutans* clinical isolates is not associated with genetic diversity within the *scrA* gene.

## Additional files

Additional file 1: A schematic representation of *scrA* gene on the chromosome of UA159, the genome sequence reference strain, with the location of the primer pairs used for PCR amplification. (JPG 381 kb)

Additional file 2: A typical DNA agarose gel showing the PCR bands obtained with the five different primer pairs used to amplify the *scrA* gene in S-ECC and CF vs. UA159 as positive control. We can see all the primers can amplify the target product. (JPG 1054 kb)

Additional file 3: As shown in Additional file 3, the nucleotide and aa sequences of scrA locus of UA159 were presented. i) ScrA active sites (568T, 570H, 585H, 587G) are highlighted in blue color box. ii) Phosphorylation sites (24H, 585H) are highlighted in red color box. iii) Hpr interaction sites (533D,534P, 535V, 536F, 540A, 541M,563Q, 564I, 566F, 567D, 573G, 574I, 575K, 581E, 582I, 583L, 585H, 589D, 591V, 592S, 604A, 636T, 639A) are highlighted in orange color box. iv) EIIB and EIIC domain are located at 2-474 amino acids, EIIA domain is at 517-640 amino acids. The missense mutations (codon 10, 1487, 1822, and 1863, red color) we found were not located in any above specific domains (red in word). (JPG 3356 kb)

## Abbreviations

ABC Transporter: ATP-binding cassette transporters
CF: Caries free
CV: Crystal violet
DNA: Deoxyribonucleic acid
ECM: Extracellular matrix
EI: Enzyme I
EII: Enzyme II
FTF: Fructosyltransferase
GTF: Glucosyltransferases
HPr: Heat-stable protein
Msm: Multiple-sugar metabolism system
PCR: Polymerase chain reaction
PTS: Phosphoenolpyruvate:sugar phosphotransferase system
*S. mutans*: *Streptococcus mutans*
S-ECC: Severe early childhood caries
