# Supplementary figures and images for: Genetic polymorphism of scrA gene of Streptococcus mutans isolates is not associated with biofilm formation in severe early childhood caries
Source: BMC Oral Health. 2017 Jul 14;17:114. doi: 10.1186/s12903-017-0407-0 (PMC5513023; doi:10.1186/s12903-017-0407-0)

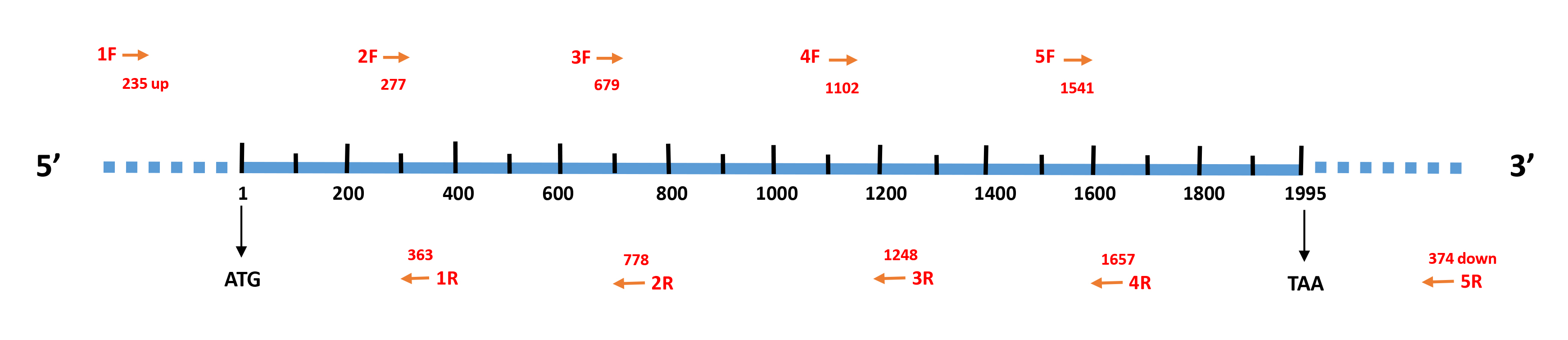

Supplement: Supplementary file 1 — A schematic representation of scrA gene on the chromosome of UA159, the genome sequence reference strain, with the location of the primer pairs used for PCR amplification. (JPG 381 kb) [file 12903_2017_407_MOESM1_ESM.jpg]

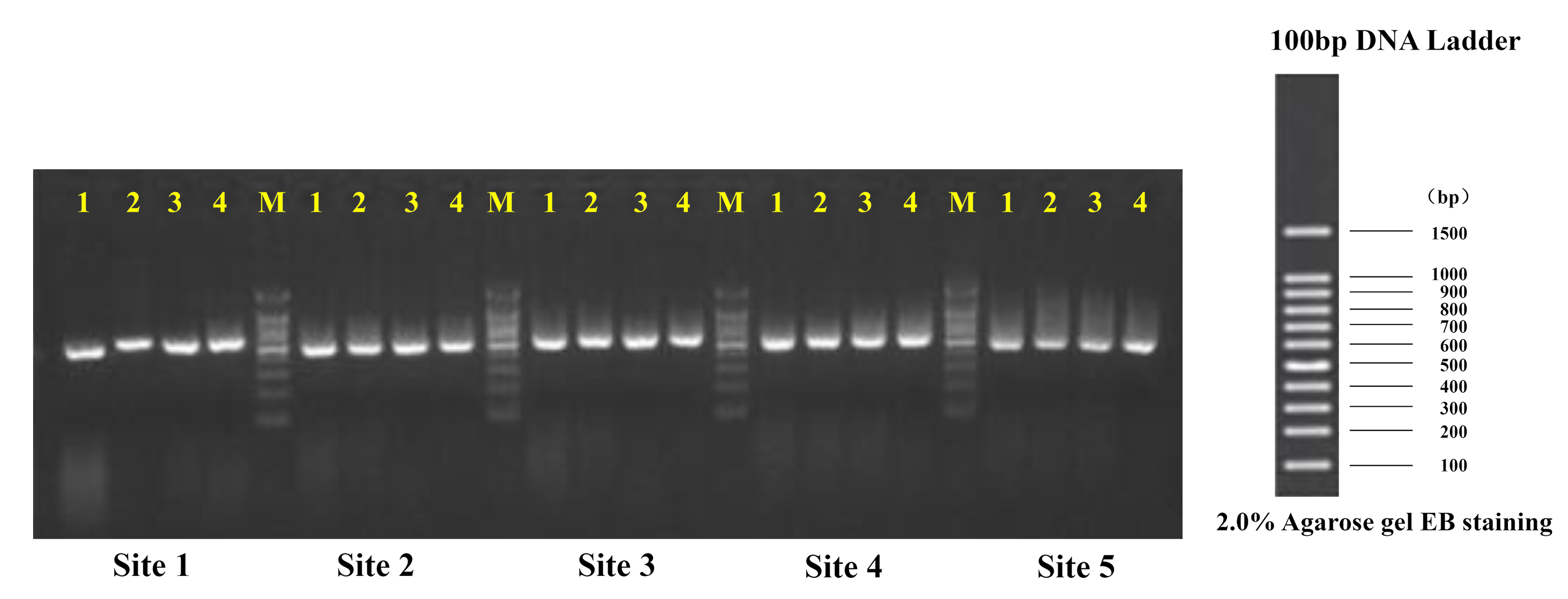

Supplement: Supplementary file 2 — A typical DNA agarose gel showing the PCR bands obtained with the five different primer pairs used to amplify the scrA gene in S-ECC and CF vs. UA159 as positive control. We can see all the primers can amplify the target product. (JPG 1054 kb) [file 12903_2017_407_MOESM2_ESM.jpg]

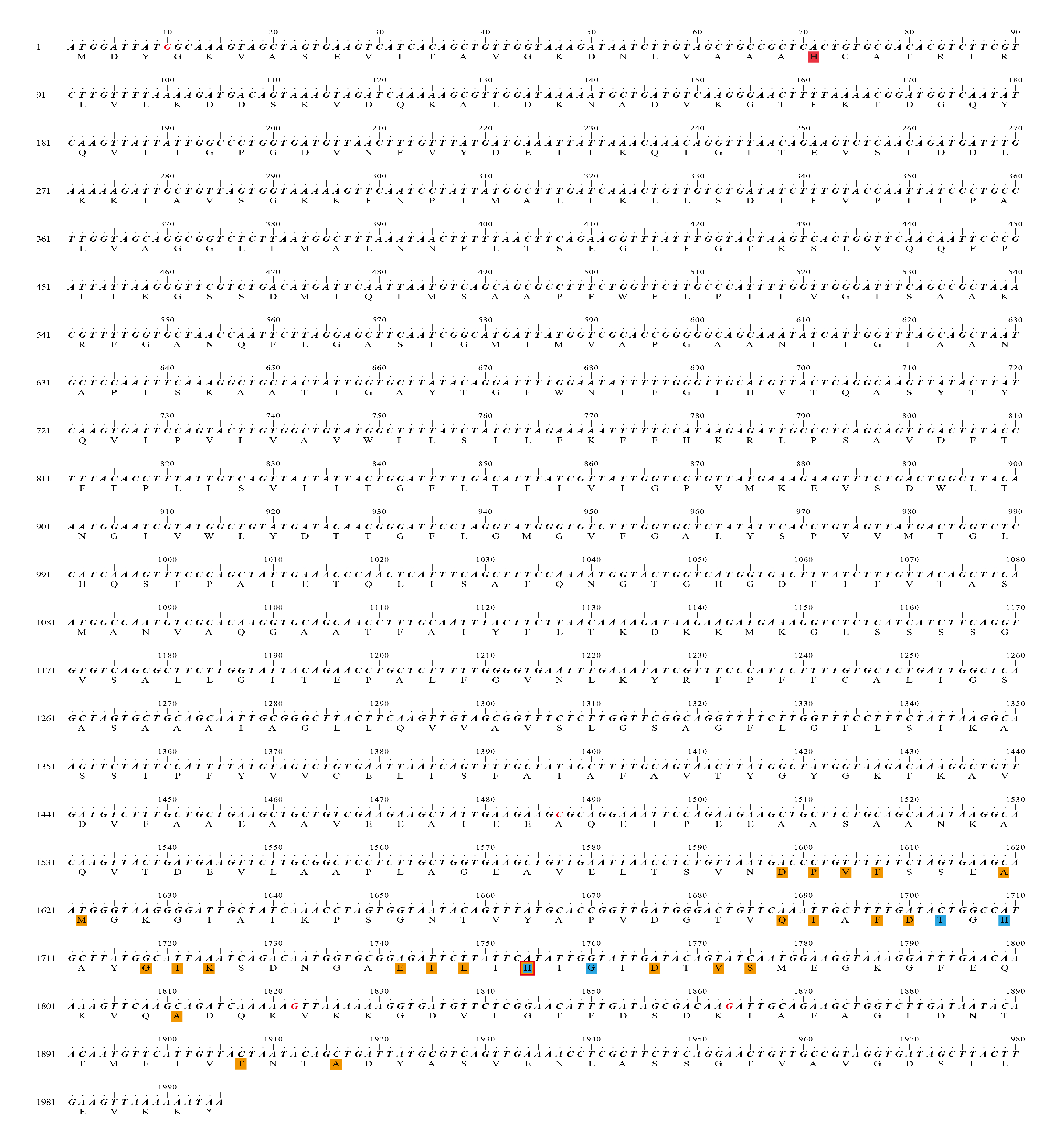

Supplement: Supplementary file 3 — As shown in Additional file 3, the nucleotide and aa sequences of scrA locus of UA159 were presented. i) ScrA active sites (568T, 570H, 585H, 587G) are highlighted in blue color box. ii) Phosphorylation sites (24H, 585H) are highlighted in red color box. iii) Hpr interaction sites (533D,534P, 535V, 536F, 540A, 541M,563Q, 564I, 566F, 567D, 573G, 574I, 575K, 581E, 582I, 583L, 585H, 589D, 591V, 592S, 604A, 636T, 639A) are highlighted in orange color box. iv) EIIB and EIIC domain are located at 2-474 amino acids, EIIA domain is at 517-640 amino acids. The missense mutations (codon 10, 1487, 1822, and 1863, red color) we found were not located in any above specific domains (red in word). (JPG 3356 kb) [file 12903_2017_407_MOESM3_ESM.jpg]
